# Blockade of glutamine-dependent cell survival augments antitumor efficacy of CPI-613 in head and neck cancer

**DOI:** 10.1186/s13046-021-02207-y

**Published:** 2021-12-14

**Authors:** Liwei Lang, Fang Wang, Zhichun Ding, Xiangdong Zhao, Reid Loveless, Jin Xie, Chloe Shay, Peng Qiu, Yonggang Ke, Nabil F. Saba, Yong Teng

**Affiliations:** 1 Department of Oral Biology and Diagnostic Sciences, Augusta University, Augusta, GA 30912 USA; 2 Georgia Cancer Center, Augusta University, Augusta, GA 30912 USA; 3 Department of Chemistry, University of Georgia, Athens, GA 30602 USA; 4 Department of Pediatrics, Emory Children’s Center, Emory University, Atlanta, GA 30322 USA; 5 Department of Biomedical Engineering, Georgia Institute of Technology and Emory University, Atlanta, 30332 USA; 6 Department of Hematology and Medical Oncology, Winship Cancer Institute, Emory University, School of Medicine, 201 Dowman Dr, Atlanta, GA 30322 USA

**Keywords:** CPI-613, GLS1, CB-839, Glutaminolysis, Combined targeting, HNSCC

## Abstract

**Background:**

Alterations in metabolism are one of the emerging hallmarks of cancer cells and targeting dysregulated cancer metabolism provides a new approach to developing more selective therapeutics. However, insufficient blockade critical metabolic dependencies of cancer allows the development of metabolic bypasses, thus limiting therapeutic benefits.

**Methods:**

A series of head and neck squamous cell carcinoma (HNSCC) cell lines and animal models were used to determine the efficacy of CPI-613 and CB-839 when given alone or in combination. Glutaminase 1 (GLS1) depletion was achieved by lentiviral shRNAs. Cell viability and apoptosis were determined in HNSCC cells cultured in 2D culture dish and SeedEZ™ 3D scaffold. Molecular alterations were examined by Western blotting and immunohistochemistry. Metabolic changes were assessed by glucose uptake, lactate production, glutathione levels, and oxygen consumption rate.

**Results:**

We show here that HNSCC cells display strong addiction to glutamine. CPI-613, a novel lipoate analog, redirects cellular activity towards tumor-promoting glutaminolysis, leading to low anticancer efficacy in HNSCC cells. Mechanistically, CPI-613 inhibits the tricarboxylic acid cycle by blocking the enzyme activities of pyruvate dehydrogenase and alpha-ketoglutarate dehydrogenase, which upregulates GLS1 and eventually promotes the compensatory role of glutaminolysis in cancer cell survival. Most importantly, the addition of a GLS1 inhibitor CB-839 to CPI-613 treatment abrogates the metabolic dependency of HNSCC cells on glutamine, achieving a synergistic anticancer effect in glutamine-addicted HNSCC.

**Conclusions:**

These findings uncover the critical role of GLS1-mediated glutaminolysis in CPI-613 treatment and suggest that the CB-839 and CPI-613 combination may potentiate synergistic anticancer activity for HNSCC therapeutic gain.

**Supplementary Information:**

The online version contains supplementary material available at 10.1186/s13046-021-02207-y.

## Background

Cancer cells maintain distinctive energy metabolism networks to support and enable cell survival, growth, progression, and metastasis under harsh conditions [1, 2]. Although there are many metabolic pathways and processes enable cancer cells to manage metabolic stress, the lipoate-sensitive regulatory process, in particular, is systematically redesigned in cancer cells to alter the metabolic status of the mitochondrial matrix and increase metabolic fluxes [3–5]. Consequently, the major enzymes involved in the lipoate-sensitive regulatory process represent valuable targets for chemotherapeutic intervention [6]. CPI-613 is a novel lipoate analog that functions to inhibit tumor mitochondrial metabolism by simultaneously attacking the tricarboxylic acid (TCA) cycle enzymes pyruvate dehydrogenase (PDH) and alpha-ketoglutarate dehydrogenase (α-KGDH) [3, 7].

Although the mechanism of action of CPI-613 appears distinct from that of the standard classes of anticancer agents used in the clinic, it clearly demonstrates both in vitro and in vivo anticancer activity [8]. The anticancer effect of CPI-613 has been evaluated in xenograft models of pancreatic cancer [9] and applied in patients with metastatic pancreatic cancer, where it has shown strong tumor growth inhibition at its maximum tolerated dose of 500 mg/m^2^ [10]. Our group recently identified that the upregulation of 5′ AMP-activated protein kinase (AMPK)-acetyl-coenzyme A carboxylase (ACC) signaling rewires lipid metabolism when pancreatic cancer cells are treated with CPI-613, which can be favorable to reactive oxygen species (ROS)-associated apoptosis [7]. In addition, CPI-613 was reported to preferentially target ovarian cancer stem cells and augment the impact of current treatment strategies on extending either progression-free or overall survival in ovarian cancer patients [11]. However, whether CPI-613 exhibits anticancer potential in other types of cancer, including head and neck squamous cell carcinoma (HNSCC), has not yet been determined.

Cancer cells rely heavily on elevated glutaminolysis to catabolize glutamine and, in turn, generate ATP and lactate to maintain TCA cycle function and compensate for metabolic alterations [12, 13]. Through its donation of nitrogen to purines and pyrimidines, glutamine performs an essential role in nucleotide biosynthesis and has been established as a second principal growth-supporting substrate [12]. The deamination of glutamine by glutaminase (GLS) within the mitochondria yields glutamate and ammonia and, importantly, marks the first step of glutaminolysis, which contributes to tumor growth by promoting cell proliferation and repressing cell death [12, 14]. In humans, the GLS family is composed of two key members: GLS1 and GLS2 [15]. While the expression of GLS2 is commonly repressed in cancers, GLS1 is typically upregulated in cancers, making it an appealing target for cancer therapies [12, 16–18]. As the gatekeeper of glutaminolysis, GLS has accordingly garnered significant interest for its potential to suppress tumor progression and metastasis. However, a better understanding of the molecular regulation of GLS in cancer cells is required to fully reveal the potential applications of GLS-based treatment.

HNSCC is the sixth prevalent cancer worldwide. Unfortunately, the clinical benefits of current HNSCC treatments, such as FDA-approved PD1 inhibitors (pembrolizumab and nivolumab), the epidermal growth factor receptor (EGFR) monoclonal antibody (cetuximab), and conventional platinum-based chemotherapy, remain unsatisfactory for patients with poor prognoses, largely due to the therapeutic evasion, resistance, and recurrence of HNSCC [19, 20]. Increasing interest in the metabolic vulnerabilities of cancer has given rise to the development of metabolism-targeted therapies that can target metabolic enzymes and pathways necessary for HNSCC malignancy. Here, we show that CPI-613 monotherapy fails to achieve a sufficient anticancer effect in HNSCC cells due to its redirection of cellular activity towards tumor-promoting glutaminolysis. Combining CPI-613 with a GLS inhibitor CB-839, however, abrogates the dependency of HNSCC cells on glutamine, representing a novel synergistic strategy to overcome glutamine-addicted HNSCC.

## Methods

### Cell culture and standard assays

Human HNSCC cell lines (HN6, HN12, HN13, HN30, and HN31) were maintained in our lab and used for experiments before passage 10. All cell lines were cultured in DMEM medium containing 10% fetal bovine serum at 37 °C in a humidified incubator with 5% CO_2_. Western blotting, plasmid transfection, lentiviral infection, cell proliferation, and colony formation assays were carried out as we previously described [7, 21, 22].

### Reagents, antibodies, and constructs

Texas-red phalloidin was purchased from Invitrogen (Carlsbad, CA). CPI-613 and CB-839 were obtained from Selleckchem (Houston, TX). Antibodies against GLS1, GLUD1, HKI, HKII, PKM1, PKM2, PFKP, PDH, α-KGDH and p-PDHA1 (Ser293) were purchased from Cell Signaling Technology (Beverly, MA). 3-(4,5-Dimethylthiazol-2-yl)-2,5-diphenyltetrazolium bromide (MTT) and an antibody that recognizes β-Actin were obtained from Sigma-Aldrich (St Louis, MO). For gene knockdown, the pLKO.1-puro TRC control shRNA targeting the gene encoding green fluorescent protein (GFP) and shRNAs targeting the GLS1 gene were purchased from Horizon Discovery (Waterbeach, UK).

### Apoptosis detection

For cultured cells, apoptosis was assessed by flow cytometry using fluorescein isothiocyanate (FITC) Annexin V Apoptosis Detection Kit (BD Biosciences, San Jose, CA) with propidium iodide (PI). In xenograft tumor sections, apoptosis was determined by the DeadEnd™ Fluorometric TUNEL System (Promega, Madison, WI). All TUNEL-positive cells in each section were counted in twenty randomly selected fields using a fluorescence microscope (Zeiss, Oberkochen, Germany).

### PDH activity assay

The activity of PDH in HNSCC cells was measured by a colorimetric assay according to the previously described method [23]. Briefly, 1 × 10^4^ cells were seeded into 96-well plate overnight before being permeabilized with 0.5% Triton X-100. Cells were incubated in 50 mM Tris-HCl (pH 7.8) buffer supplemented with 3-bromopyruvate (5 mM), MgCl_2_ (1 mM), EDTA (0.05 mM), Triton X-100 (0.0025%), thiamine diphosphate (0.3 mM), rotenone (10 μM), pyruvate (10 mM), NAD^+^ (3 mM), Co-A (1 mM), nitroblue tetrazolium (0.75 mM) and phenazine methosulfate (0.05 mM) for 45 min at 37 °C. Insoluble blue formazan precipitates were dissolved in 10% SDS in 0.01 M HCl overnight and absorbance was monitored at OD 570 nm.

### Measurement of ATP, glucose, lactate, and glutathione (GSH)

CellTiter-Glo® 2.0 Assay Kit was used to determine the ATP amount in cell lysates. Glucose-Glo™ Assay Kit and Lactate-Glo™ Assay Kit (Promega, Madison, WI) were used to measure glucose uptake and lactate levels in cell supernatants, respectively. Glutamine/Glutamate-Glo Assay (Promega) was used to assess the changes in glutamine and glutamate content in cells with indicated treatments. Changes in the level of total intracellular GSH were determined using Glutathione Detection Assay Kit (Fluorometric, Abcam, MA). For GSH determination, HN6 and HN31 cells were treated with or without 100 μM CPI-613 for 24 h, and the lysates were collected and incubated on ice for 30 min. The supernatants were collected after centrifugation at 12,000×g for 10 min and processed according to the kit protocol, and fluorescence was measured at 380/461 nm (excitation/emission wavelengths).

### Mitochondrial respiratory assays and metabolite determination

Oxygen consumption rate (OCR) was measured using Seahorse XF Cell Energy Phenotype Test Kit on a Seahorse XF96 Extracellular Flux analyzer (Agilent Technology). Briefly, 4 × 10^3^ cancer cells were seeded on each well of poly-D-lysine coated XF96 miniplates and cultured in DMEM containing 10% FBS overnight, then kept in 100 μl XF medium for 45 mins (XF base medium containing 10 mM glucose, 2 mM L-glutamine, and 1 mM sodium pyruvate). Three OCR measurements were obtained under basal conditions and upon sequential injection of 1 μM oligomycin (Oligo) and 1 μM fluoro-carbonyl-cyanide phenylhydrazone (FCCP). OCR values were calculated from 3-min measurement cycles and adjusted to cell numbers.

### Tumorsphere culture

Dissociated single HN6 and HN31 cells were plated on 6-well ultra-low attachment plates (Corning, New York, NY) at a density of 1 × 10^5^ cells per ml and grown in serum-free DMEM/F-12 medium supplemented with B27 (Invitrogen, Waltham, MA), 20 ng/ml epidermal growth factor (EGF), 20 ng/ml basic fibroblast growth factor (b-FGF), and 5 μg/ml insulin (Peprotech, Rocky Hill, NJ). Two weeks after incubation, the numbers of spheres were counted using a Zeiss microscope.

### Three-dimensional (3D) cell culture

Approximately 1.0 × 10^5^ HNSCC cells were seeded into SeedEZ™ scaffold (Lena Bioscience, Atlanta, GA) for 7 days before treatment with CPI-613 and CB-839 alone or in combination. After one-week of drug treatment, cell viability in SeedEZ™ scaffold was measured by alamarBlue Cell Viability Reagent (Thermo Fisher Scientific, Waltham, MA) at 545/590 nm ex/em, followed by staining with Texas-red phalloidin and imaging.

### Animal studies, drug administration and immunohistochemistry (IHC)

Six-week-old NOD.Cg-*Prkdcscid Il2rgtm1Wjl/SzJ* (NSG) mice were purchased from the Jackson Laboratory (Bar Harbor, ME). An orthotopic tongue tumor model was generated as we previously described [22, 24]. To determine the function of GLS1, 1 × 10^5^ GLS1 knockdown and control HN6 cells were suspended in 50 μl of PBS/Matrigel (3:1) and injected into the anterior ~ 1/3 tongue of NSG mice under anesthesia. To determine the therapeutic efficacy of single or combined drug treatment 7 days after HN6 cell implantation, tumor-bearing mice were randomized into four groups to receive vehicle (PBS), CPI-613, CB-839, or the combination of CPI-613 and CB-839 for a total of 2 weeks. CPI-613 was administered by intraperitoneal injection (*i.p.*) every 3 days at the dose of 25 mg/kg body weight, and CB-839 was administered by oral gavage twice a day at the dose of 200 mg/kg body weight. Mice were imaged for bioluminescent luciferase signal every week using a Xenogen IVIS-200 In Vivo Imaging System (PerkinElmer, Waltham, MA). When experiments were terminated, primary tongue xenografts and major organs (including the heart, intestine, kidney, liver, and lung) from the mice were excised and processed for H&E staining and IHC. Tumor volume measurement was performed by digital caliper according to the formula V = length × width^2^ × 1/2. If the mice were implanted with luciferase-expressing cancer cells, tumor burden was assessed using bioluminescence imaging. All animal experiments were approved by the Institutional Animal Care and Use Committee (IACUC) of Augusta University. Sections of paraffin-embedded mouse tongue tumors that received different treatments were deparaffinized and rehydrated by standard pathology laboratory methods, followed by heat-induced antigen retrieval and IHC with anti-Ki67 antibody (Abcam, Cambridge, UK). The sections were developed with DAB substrate kit (Vector Laboratories, Burlingame, CA) and counterstained with hematoxylin. IHC staining was quantified using Image pro-Plus6.0 software (Media Cybernetics, Silver Springs, MD, USA) and presented as integrated optical density (IOD).

### The Cancer genome atlas (TCGA) data retrieval and statistical analysis

Data on GLS1 gene expression for 33 cancer types and adjacent non-carcinoma tissues were extracted from TCGA (https://tcga.xenahubs.net) and used to generate an expression matrix. Hazard ratios (HRs) from TCGA pan-cancer data were used to measure the prognostic significance of GLS1*.* Student’s *t-*test was used for comparison of two groups and analysis of variance (ANOVA) was used for comparison of multiple groups. The data obtained from three independent repetitions were presented in the form of an average and a standard deviation. All statistical calculation graphs were generated by GraphPad Prism 9.0 and a *p*-value of < 0.05 was set as the criterion for statistical significance. To assess the drug combination effect, combination index (CI) values were calculated using the software ComboSyn [25]. CI values of less than 1, equal to 1, and greater than 1 indicated synergistic, additive, and antagonistic effects, respectively.

## Results

### CPI-613-induced GLS1 upregulation abrogates CPI-613 cytotoxicity in HNSCC cells

**Fig. 1:**
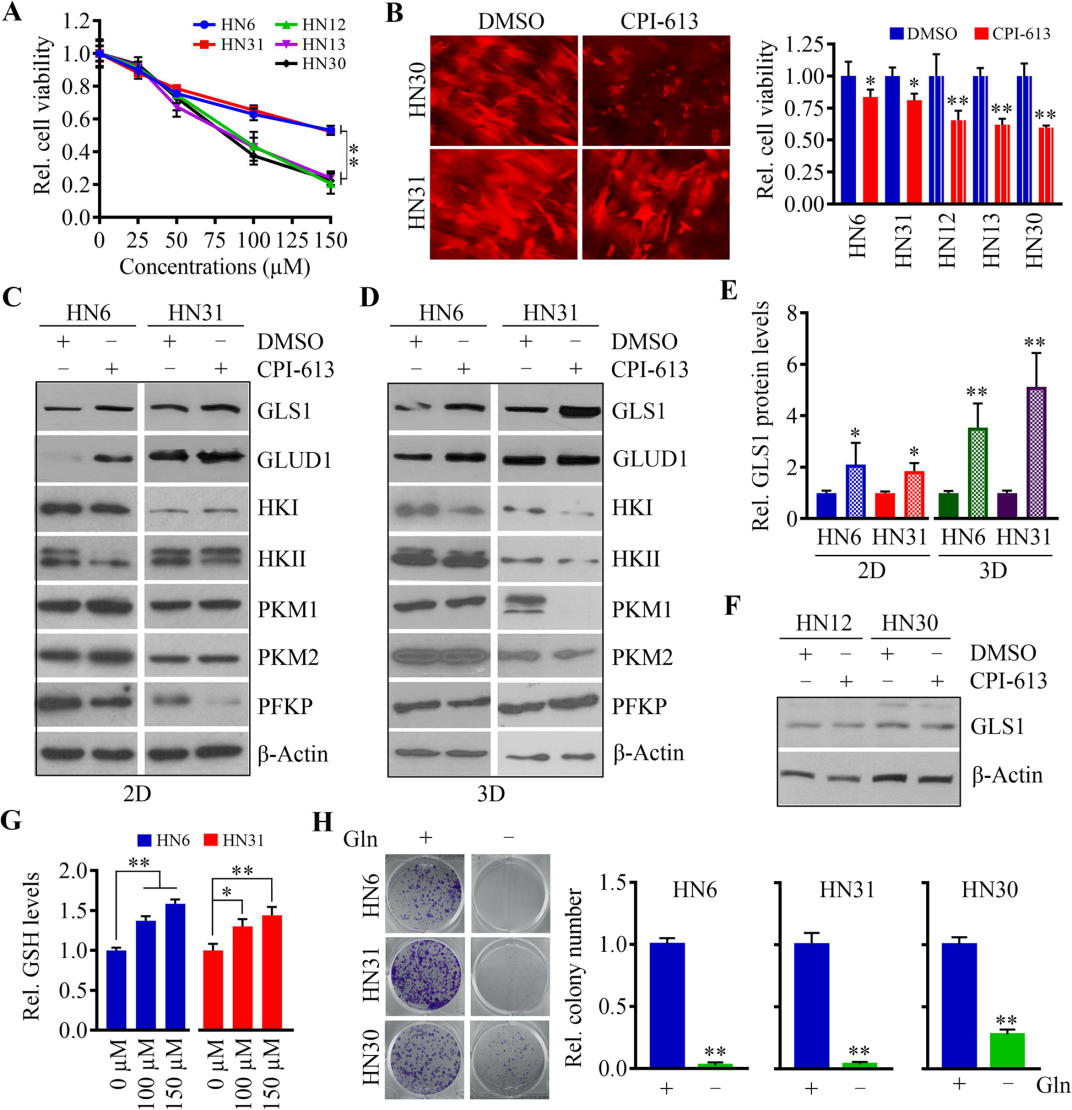
CPI-613-induced GLS1 upregulation abrogates CPI-613 cytotoxicity in HNSCC cells. **A** The effect of CPI-613 on HNSCC cell viability determined by MTT after a three-day treatment. **B** The effect of CPI-613 on HNSCC cell viability in SeedEZ™ 3D scaffold after a fourteen-day treatment. Representative fluorescence images from HN30 and HN31 cells and quantitative data from three independent experiments are shown in the left and right panels, respectively. **C**-**E** The effect of CPI-613 on the protein levels of glycolytic and glutamine metabolism-related enzymes. Cell lysates collected from 2D culture dish (**C**) and SeedEZ™ 3D scaffold (**D**) were subjected to this analysis. Representative Western blotting images and quantitative data from three independent experiments are shown in (**C**, **D**) and (**E**), respectively. **F** The effect of CPI-613 on GLS1 levels in CPI-613 sensitive cell lines, HN12 and HN30. **G** The effect of CPI-613 on GSH level in HNSCC cells determined by a Glutathione Detection Assay Kit. (H) The effect of glutamine (Gln) on HNSCC cell viability determined by a colony formation assay. *p < 0.05, **p < 0.01

To determine the anti-HNSCC effect of CPI-613, five HNSCC cell lines (HN6, HN12, HN13, HN30, and HN31) were treated with varying doses of CPI-613 for 3 days. Although all cell lines examined showed dose-dependent decreases in cell viability, HN6 and HN31 were more resistant to CPI-613 treatment than the other three cell lines (Fig. 1A). The TCA cycle metabolizes acetate derived from carbohydrates, proteins, and fats to generate ATP [26, 27]. As CPI-613 treatment resulted in significant reduction of metabolic flux through the TCA cycle [7], we asked whether CPI-613 could block ATP production in HNSCC cells. As shown in Supplementary Fig. S1, CPI-613 reduced the amount of ATP in both cell lines cultured in either glucose or pyruvate, but the reduction in ATP amount in HN6 cells was less than that in HN12 cells under the same conditions. This result was consistent with the effect of CPI-613 on proliferation of these two cell lines (Fig. 1A). Because 3D cell cultures more faithfully recapitulate the complex aspects of cancer growth and drug response in vitro [28], we also cultured HNSCC cells in SeedEZ™ 3D scaffolds, which are composed of completely inert and transparent glass microfibers, for 7 days before CPI-613 treatment. In line with the results from traditional 2D culture, HN6 and HN31 were more resistant to CPI-613 than the other cell lines examined in this study (Fig. 1B). One possible reason for the low cytotoxicity of CPI-613 in certain HNSCC cells was that the targeting of PDH/α-KGDH redirected cell growth and survival to other metabolic pathways. To assess this possibility, we determined the changes in metabolic enzymes in HN6 and HN31 cells in the presence or absence of CPI-613. This analysis identified that GLS1, a mitochondrial enzyme that hydrolyzes glutamine into glutamate to fuel rapid cancer cell proliferation, was the only molecule upregulated in both CPI-613-treated HN6 and HN31 cells, regardless of their growth in 2D culture dishes or SeedEZ™ scaffolds (Fig. 1C-E), suggesting that glutaminolysis plays a compensatory role in cell survival upon CPI-613 treatment. GLUD1 was also upregulated in CPI-613-treated 2D cells; however, it remained at the same levels in cells cultured in 3D cultures with or without CPI-613 treatment (Fig. 1D). These findings motivated our further study of GLS1. Next, we determined GLS1 levels in two CPI-613 sensitive cell lines, HN12 and HN30, in the presence and absence of CPI-613. GLS1 levels were unchanged in these two cell lines treated with and without CPI-613 (Fig. 1F), indicating that upregulation of GLS1 is one major mechanism associated with low treatment efficacy of CPI-613.

Glutamine is an effective precursor of glutamate for GSH synthesis in many cell types, including cancer cells [29]. Consistent with this notion, increased GSH levels were observed in CPI-613 treated HN6 and HN31 cells (Fig. 1G), indicating that HNSCC cells experience enhanced glutaminolysis upon CPI-613 treatment. To determine whether HNSCC cells are glutamate-dependent, we cultured HN6, HN30, and HN31 cells in medium with or without 4 mM glutamate. Colony formation assay showed that glutamate deprivation impaired the proliferation of all three HNSCC cell lines (Fig. 1H), but HN6 and HN31 cells were more addicted to glutamate than HN30 cells (Fig. 1H), which may explain their greater resistance to CPI-613 treatment.

### GLS1 sustains HNSCC cell growth and survival by upregulating glutamine metabolism

**Fig. 2:**
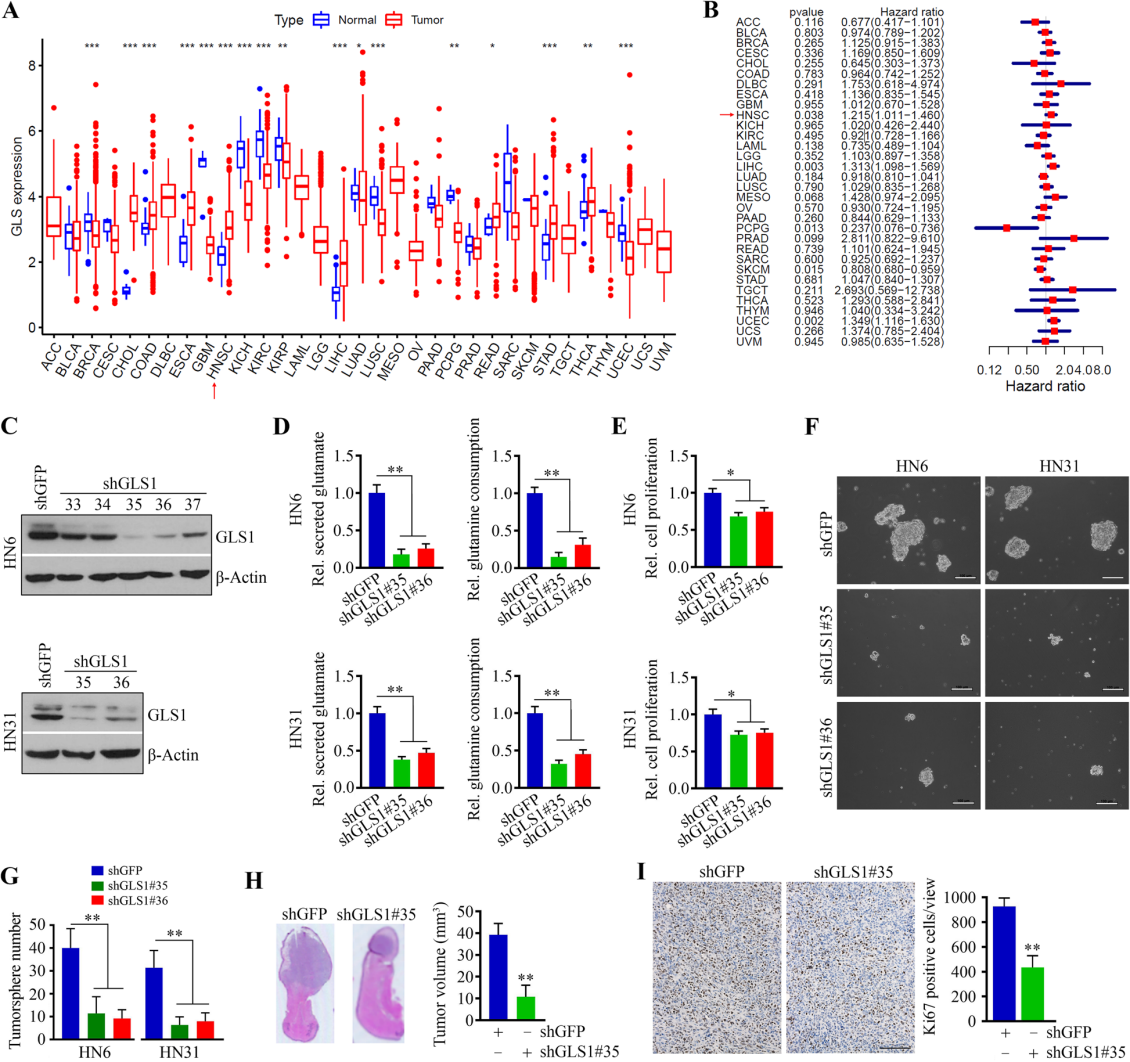
GLS1 sustains HNSCC cell growth and survival by upregulating glutamine metabolism. **A** The boxplot shows the pan-cancer expression profiling of GLS1 in human cancers. The row below refers to the standard abbreviations of tumors in TCGA cohorts. **B** Forest plot visualizing the HRs of GLS1 in human cancers. The first three columns display the cancer type, *p*-value, and HR, respectively. In the forest plot, risk factors are shown in red. (**C**) The knockdown effect of shGLS1 in HNSCC cells determined by Western blotting. shGFP: a negative control shRNA targeting the gene encoding GFP; shGLS1: shRNAs targeting the GLS1 gene. Numbers indicate different sequences of shGLS1. **D** Changes in secreted glutamate and glutamine consumption determined by Glutamine/Glutamate-Glo Assays in HNSCC cells with or without GLS1 knockdown. **E** The effect of GLS1 knockdown on HNSCC cell viability determined by MTT. **F**, **G** The effect of GLS1 knockdown on tumorsphere formation in HN6 and HN12 cells. Representative images and quantitative are shown in (**F**) and (**G**), respectively. Scale bar = 100 μm. **H** The effect of GLS1 knockdown on HN6-derived tongue tumor growth in NSG mice. Representative tongue tumors and quantitative data of tumor volume (*n* = 5/group) are shown in the upper and lower panels, respectively. **I** The effect of GLS1 knockdown on tumor cell proliferation determined by IHC with anti-Ki67 antibody. Representative IHC images and quantitative data (*n* = 5) are shown in left and right panels, respectively. Scale bar = 50 μm. *p < 0.05, **p < 0.01

TCGA comprises over 20,000 samples from 33 types of cancer and corresponding non-carcinoma samples. Using this data, we found that GLS1 expression is elevated in HNSCC tissues relative to matched non-carcinoma tissues (Fig. 2A). We then integrated HRs for overall survival in the form of a meta-analysis to assess the prognostic value of GLS1 score in patients cross cancer types. Increased GLS1 risk score correlated with overall survival in HNSCC patients [HR: 1.215, 95% confidence interval (CI): 1.011–1.460, *p* = 0.038] (Fig. 2B), suggesting that GLS1 is a prognosis-associated gene for this cancer type. High levels of GLS1 also indicate poor patient survival in liver hepatocellular carcinoma (LIHC, HR: 1.313, 95% CI: 1.098–1.569, *p* = 0.003) and uterine corpus endometrial carcinoma (UCEC, HR: 1.349; 95% CI: 1.116–1.630, *p* = 0.002). To interrogate the role of GLS1 in HNSCC cells, we depleted its expression in HN6 and HN31 cells using shRNAs (Fig. 2C). Compared with the knockdown controls, GLS1 depletion dramatically decreased the levels of secreted glutamate and reduced glutamine uptake (Fig. 2D), leading to repression of HNSCC cell proliferation (Fig. 2E). Moreover, GLS1 knockdown in both HN6 and HN31 cells reduced the efficiency of tumorsphere formation as evidenced by lower number and smaller size of tumorspheres relative to the knockdown control cells (Fig. 2F and G). This result is consistent with a previous report regarding the function of GLS1 in hepatocellular carcinoma [30], supporting the role of GLS1 in promoting HNSCC cell stemness.

To validate these observations in animals, GLS1 knockdown and its control HN6 cells were individually injected into the anterior tongue of NSG mice to generate an orthotopic tongue tumor mouse model (*n* = 5/group). Four weeks after inoculation, a marked decrease in tumor volume, along with a significant reduction in Ki67 positive tumor cells, were observed in mice implanted with GLS1 knockdown cells (Fig. 2H and I), We also performed TUNEL assays to determine apoptosis; however, no significant change was seen in apoptotic rate with or without GLS1 loss in xenograft tumors (data not shown). These data support the pivotal role of GLS1 in sustaining HNSCC cell growth.

### Loss of GLS1 sensitizes HNSCC cells to CPI-613

**Fig. 3:**
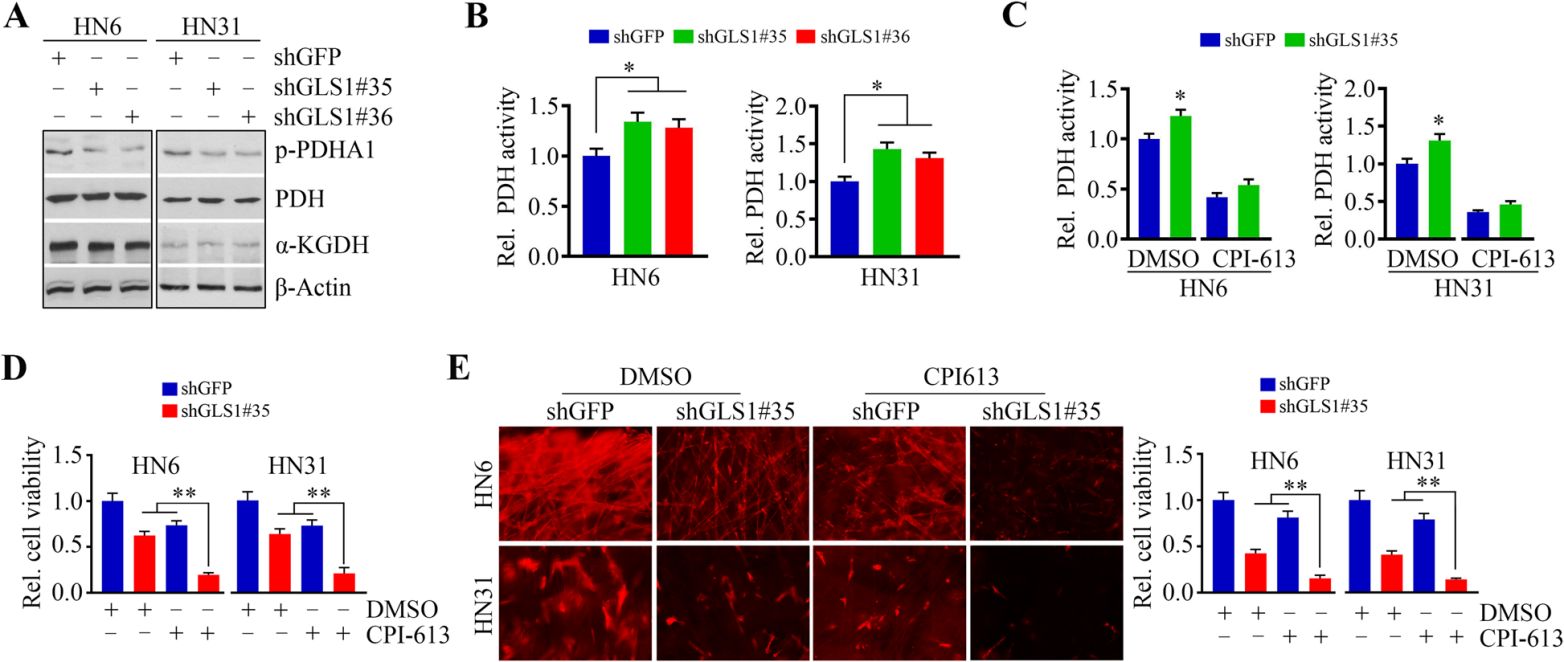
GLS1 depletion sensitizes HNSCC cells to CPI-613. **A** The effect of GLS1 knockdown on PDH and α-KGDH protein levels determined by Western blotting. **B** The effect of GLS1 knockdown on PDH activity in HNSCC cells. **C** The effect of combined GLS1 knockdown and CPI-613 treatment on PDH activity in HNSCC cells. **D** The effect of combined GLS1 knockdown and CPI-613 treatment on HNSCC cell viability. **E** The effect of combined GLS1 knockdown and CPI-613 treatment on HNSCC cell viability in SeedEZ™ 3D scaffold on Day 14 after treatment. Representative fluorescence images and quantitative data from three independent experiments are shown in the left and right panels, respectively. *p < 0.05, **p < 0.01

To determine the functional association of GLS1 with cellular energy metabolism, we determined changes in the levels of PDH and α-KGDH in GLS1 knockdown and its control HN6 and HN12 cells. GLS1 depletion decreased p-PDHA1 levels but did not affect its total protein levels or α-KGDH (Fig. 3A). As the enzymatic activity of PDH is negatively regulated by phosphorylation at several serine sites [31], we then determined PDH activity in HN6 and HN12 cells with or without GLS1 knockdown. As shown in Fig. 3B, loss of GLS1 increased PDH activity in both cell lines, and this increase was significantly abrogated by additional CPI-613 treatment (Fig. 3C). These findings prompted us to study the effect of CPI-613 in combination with GLS1 knockdown in HNSCC cells. Compared with GLS1 depletion or CPI-613 treatment alone, a greater inhibitory effect on cell viability was seen when GLS1 knockdown cells were treated with CPI-613 (Fig. 3D). The same results were obtained in HN6 and HN31 cells cultured in SeedEZ™ scaffold (Fig. 3E), suggesting that synergistic blockade of glutaminolysis and energy metabolism results in a superior cytotoxic effect in HNSCC cells.

### The glutaminase inhibitor CB-839 induces tongue tumor shrinkage

**Fig. 4:**
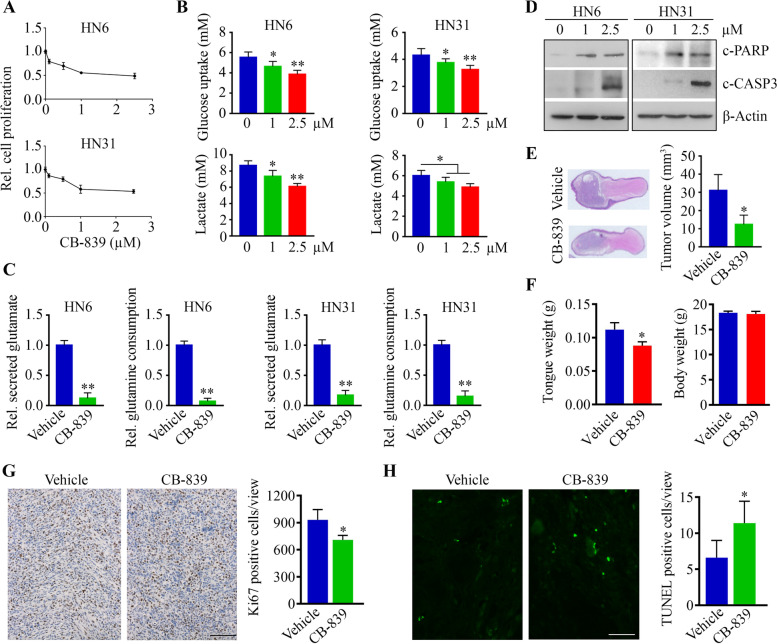
CB-839 induces the repression of tongue tumor growth. **A** The effect of CB-839 on HNSCC cell proliferation after a three-day treatment. **B** The effect of CB-839 on glucose uptake and lactate release in HNSCC cells. **C** Changes in secreted glutamate and glutamine consumption were determined by Glutamine/Glutamate-Glo Assays in HNSCC cells with or without CB-839 treatment. **D** The effect of CB-839 on apoptosis determined by Western blotting. **E**, **F** The effect of CB-839 treatment on HN6-derived tongue tumor growth in NSG mice. Representative tongue tumors and quantitative data of tumor volume, tongue weight, and body weight (*n* = 5/group) are shown in different panels. (G, H) Ki67- and TUNEL-positive tumor cells in tongue xenografts of mice receiving CB-839 or vehicle treatment. Representative images and quantitative data (*n* = 5) are shown in left and right panels, respectively. Scale bar = 50 μm. *p < 0.05, **p < 0.01

CB-839 is the first-in-clinic and orally bioavailable noncompetitive inhibitor of GLS1 splice variants, kidney-type (KGA) and glutaminase C (GAC), which convert glutamine into glutamate. There is highly credible evidence indicating the significant anti-proliferative activity of CB-839 in many types of cancers [32–35], including lung adenocarcinoma, chondrosarcoma, liver cancer, and lymphoma. However, it is still unclear whether CB-839 possesses therapeutic potential in HNSCC. To address this, we first evaluated the effect of CB-839 on cell proliferation. As shown in Fig. 4A, CB-839 dose-dependently inhibited HN6 and HN31 cell growth with an IC50 of around 1 μM. Decreased glucose uptake and lactate levels were also seen in these cells when treated with CB-839 (Fig. 4B), suggesting the effect of CB-613 in coupling glutaminolysis inhibition with decreased glucose consumption. Consistent with the data from GLS1 knockdown, CB-839 led to a reduction in secreted glutamate and glutamine consumption in HNSCC cells (Fig. 4C). Moreover, CB-839 also induced apoptosis, as evidenced by the appearance of cleaved PARP and caspase 3 in cells following its administration (Fig. 4D). In HN6-derived tumor-bearing mice, two-week treatment with CB-839 did not reduce the body weight of mice but resulted in significantly smaller tumor xenografts relative to those found in vehicle-treated animals (Fig. 4E and F). Consistently, fewer Ki67-positive tumor cells along with increased apoptosis were seen in mice receiving CB-839 compared with vehicle (Fig. 4G and H). Collectively, these observations suggest that CB-839 could be a safe and potent anti-HNSCC agent.

### CB-839 and CPI-613 combination exhibits potential synergy in HNSCC cells

**Fig. 5:**
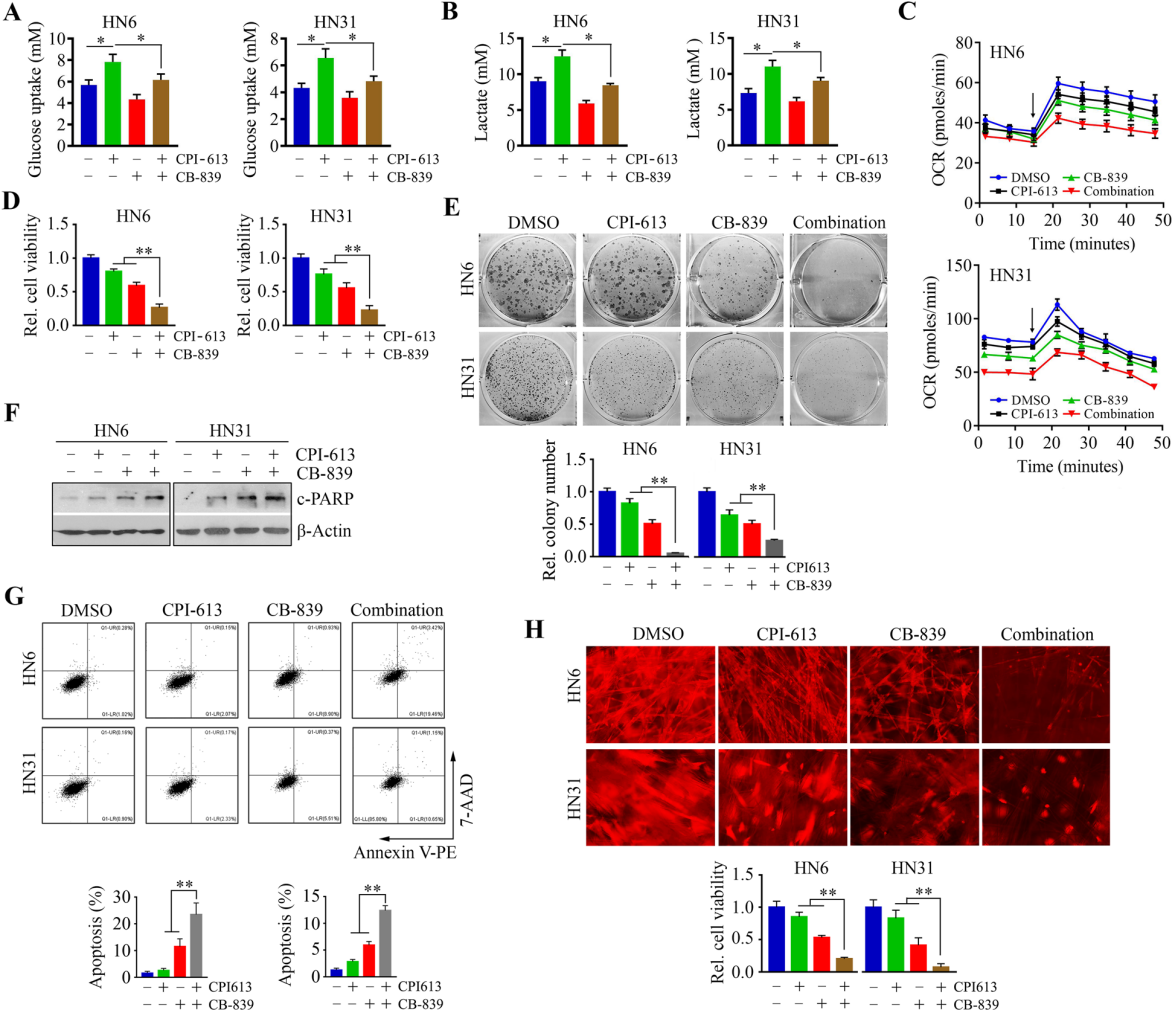
Addition of CB-839 strengthens the cytotoxic effect of CPI-613 treatment in HNSCC cells. **A**, **B** The effect of CB-839 combined with CPI-613 on glucose uptake (**A**) and lactate release (**B**) in HNSCC cells. **C** OCR profiles of HNSCC cells treated with CB-813 and CPI-613 alone or in combination, measured by a Seahorse XF analyzer. Oligo and FCCP (1.0 and 1.0 μM final, respectively) were injected at the indicated point (arrow). Notably, Oligo inhibits ATP synthesis and FCCP uncouples OXPHOS. **D** The effect of CB-839 combined with CPI-613 on HNSCC cell viability. **E** The effect of CB-839 combined with CPI-613 on cell colony formation in HNSCC cells. F, **G** The effect of CB-839 combined with CPI-613 on apoptosis determined by Western blotting (**F**) and flow cytometry (**G**). **H** The effect of CB-839 combined with CPI-613 on cell viability in SeedEZ™ 3D scaffold determined on Day 14 after treatment. In (**E**, **G**, and **H**) representative images and quantitative data from three independent experiments are shown in the upper and lower panels, respectively. *p < 0.05, **p < 0.01

The above data prompted us to assess the addition of CB-839 to CPI-613 treatment. We first investigated the role of this combination in metabolic alterations. Consistent with the enrichment in glucose utilization in cancer cells with PDH depletion [36], CPI-613 treatment alone increased glucose uptake and lactate production in both HN6 and HN31 cells (Fig. 5A and B), suggesting that inhibition of mitochondrial metabolism by CPI-613 may elicit increasing compensatory glycolytic activity. The addition of CB-839 to CPI-613 treatment attenuated the CPI-613-induced increase in glucose consumption and lactate production (Fig. 5A and B). To measure the impact of these treatments on mitochondrial metabolic function, we assessed OCR, an indicator of mitochondrial oxidative phosphorylation (OXPHOS). Remarkably, the combination of CB-839 and CPI-613 demonstrated the lowest OCR normalized to cell number among the four treatment groups (Fig. 5C). Consistent with this phenotype, co-treatment suppressed cell viability and impaired colony formation ability more efficiently than the other three treatments (Fig. 5D and E). Moreover, a strong synergistic induction of apoptosis was seen in the dual drug-treated HN6 and HN31 cells, as indicated by an increase in PARP cleavage and rates of apoptosis (Fig. 5F and G). The inhibitory effect of the CB-839 and CPI-613 combination on 3D growth was also evident compared with single-drug treatments (Fig. 5H). Taken together, these observations indicate that CB-839 augments the cytotoxic effect of CPI-613 in HNSCC cells.

### CB-839 and CPI-613 inhibit tongue tumor growth in mice more potently when combined

**Fig. 6:**
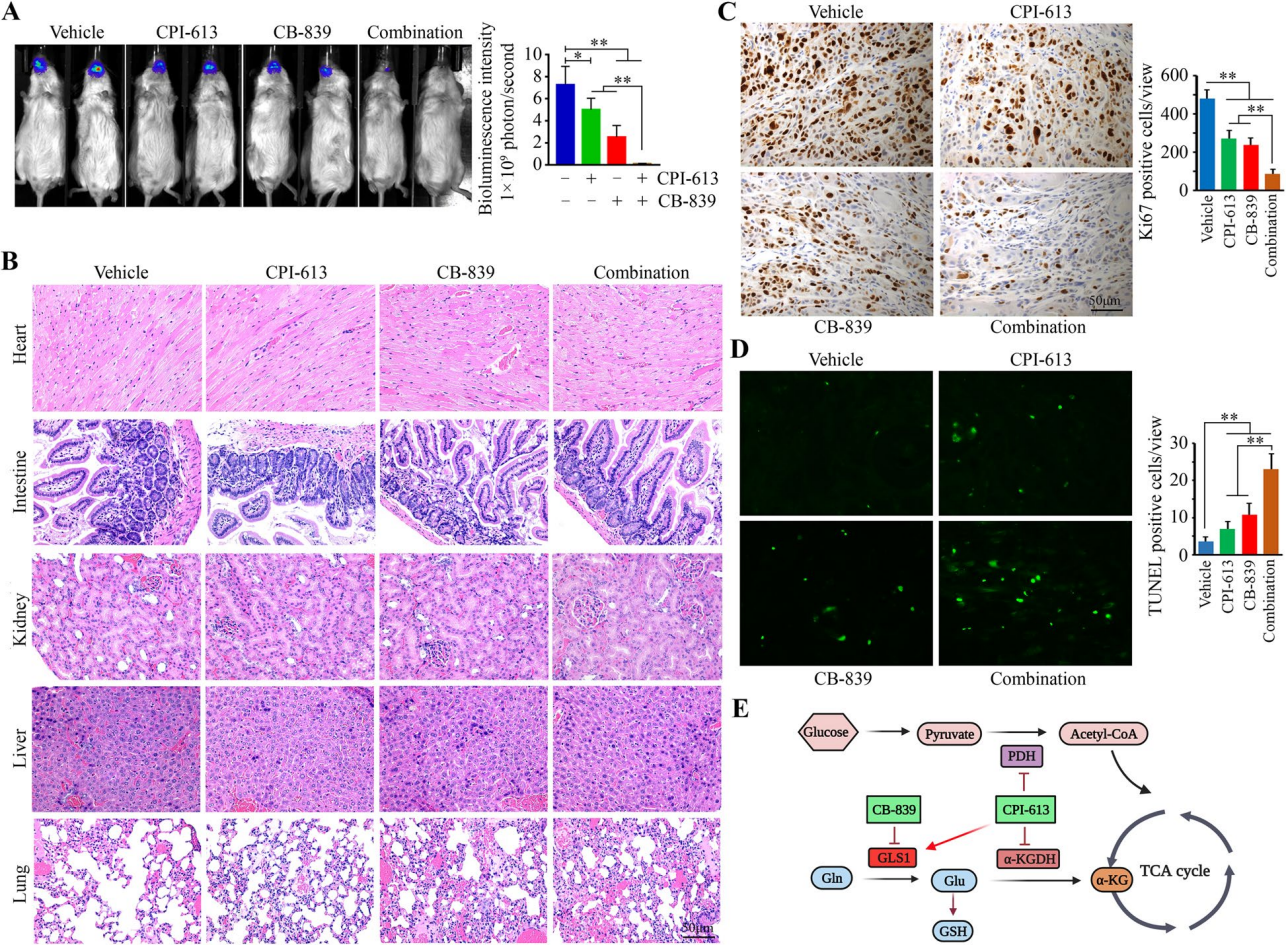
CB-839 and CPI-613 inhibit tongue tumor growth in mice more potently when combined. **A** The effects of CB-839 and CPI-613 alone or in combination on tongue tumor shrinkage on Day 14 after treatment. **B** Histology of major organs (e heart, intestine, kidney, liver, and lung) at the endpoint of each treatment. **C** Ki67 positive tumor cells in tongue xenografts of mice receiving indicated treatments determined by IHC. **D** Apoptosis in tongue xenografts of mice receiving indicated treatments determined by TUNEL assay. **E** Schematic representation of the mechanism of drug combination-mediated inhibition of head and neck tumor growth. In (**A**, **C**, **D**), representative images and quantitative data are shown in the left and right panels, respectively. *p < 0.05, **p < 0.01

To evaluate the effectiveness of the CB-839 and CPI-613 combination in vivo, luciferase-containing HN6 cells were implanted into the tongues of NSG mice to establish tumors. Mice were treated with vehicle, CB-839, CPI-613, or the combination when tongue tumors were visualized. After a two-week treatment, tumor burden was reduced in each single-arm treatment, as evidenced by lower bioluminescent signals (Fig. 6A). Most importantly, the combination of CB-839 and CPI-613 achieved an anticancer effect that was superior to either of the drug treatments alone (Fig. 6A). Histopathological analysis by H&E staining showed no morphologic changes in the major organs (Fig. 6B), suggesting this combination does not produce detectable systemic toxicities. To assess the effect of the treatments on tumor xenografts, tissue sections were immunostained with an anti-Ki67 antibody. Compared with single-arm treatment, a significant reduction in Ki67-positive cells was seen in the mice receiving the dual-drug treatment (Fig. 6C). The drug combination also led to a more robust induction of tumor cell apoptosis than single-arm treatment, as evidenced by greater numbers of TUNEL-positive cells in the tumor tissues obtained from mice in this group (Fig. 6D). These novel findings indicate that the combination of CB-839 and CPI-613 is a safe and more effective therapeutic strategy for HNSCC treatment.

## Discussion

HNSCC is one of the most commonly diagnosed cancers worldwide and remains one of the most difficult cancer types to treat due to its pathology and limited treatment options [37, 38]. Cancer cells have distinct metabolic states that support their proliferation and progression, suggesting that targeting the metabolic differences between cancer and normal cells may be a promising and novel anticancer strategy. While cancer-associated metabolic changes have been increasingly studied in recent years, very little is known about the dynamic regulation of metabolic fluxes in HNSCC development and progression. Our previous studies have provided evidence for how mitochondrial metabolic cues, including the ROS-mediated apoptotic machinery, ATP, and TCA cycle regulate HNSCC malignancy [26, 39]. In the present study, we found that targeting mitochondrial metabolism using CPI-613 as monotherapy had a very limited effect against HNSCCs with glutamine addiction. Mechanistically, CPI-613 has the potential to enhance glutaminolysis in HNSCC cells by increasing GLS1 levels (Fig. 6E). Glutaminolysis then plays a compensatory role in increasing glutamine-dependent cell survival through alternative metabolic pathways. Our findings indicate that additionally inhibiting glutaminolysis by targeting the key glutaminolysis enzyme GLS1 during CPI-613 treatment represents a more effective treatment regimen for HNSCC.

As an alpha-lipoic acid analogue, CPI-613 has been used to halt cancer cell multiplication and survival by selectively targeting PDH and α-KGDH, which are critical to the mitochondrial TCA cycle [3, 7]. This agent is a first-in-class compound targeting enzymes involved in cancer cell energy metabolism and, in combination with modified FOLFIRINOX, is currently being investigated in a Phase III clinical trial for treating patients with metastatic pancreatic cancer [40]. We provide evidence here that as a single-arm treatment, the anticancer effect of CPI-613 in HNSCCs addicted to glutamine appears to be very limited. Through molecular screening, however, we were able to identify GLS1 as the molecule critically responsible for CPI-613 sensitivity in HNSCC cells. The maintenance of a sufficient intracellular concentration of glutamate relies predominantly on the activity of GLS family members that are encoded by two different genes, GLS1 and GLS2. GLS1 overexpression was detected in HNSCC, and these higher levels are associated with lower survival in HNSCC patients [16]. Most importantly, we depleted GLS1 in HNSCC cells and found that loss of GLS1 expression led to a reduction in cell proliferation and tongue tumor growth by suppressing glutaminolysis, supporting its oncogenic role in HNSCC. Additionally, treatment with CPI-613 increased GLS1 activity, which, in turn, led to metabolic reprogramming towards higher glutaminolysis, indicating a potential metabolic vulnerability in CPI-613 use.

The levels of most metabolic enzymes we studied were unchanged changes during CPI-613 treatment regardless of culture methods. GLUD1, PFKP and PKM1 showed different responses to CPI-613 in two HNSCC cell lines or two culture methods. PFKP is the phosphofructokinase’s platelet-specific isoform modulating glycolysis [41]. An altered level of PFKP was only seen in 2D-cultured HNSCC cells in the presence of CPI-613, suggesting its drug response profiling in 3D culture is inherently different from that in 2D culture. PKM1, as well as PKM2, were only inhibited by CPI-613 in 3D cultured HN31 cells. Nonetheless, GLS1 was consistently upregulated in CPI-613-treated HN6 and HN31 cells that were seeded in 2D or 3D cultures. Recently, regulation of GLS1 and glutaminolysis in cancer has been shown to be under oncogenic control. For example, c-Myc can facilitate the increase of GLS1 expression via suppressing miR-23a/b in human P-493 B lymphoma cells and PC3 prostate cancer cells [17]. Interestingly, ErbB2 activation upregulates GLS1 expression in breast cancer cells via NF-κB pathway rather than c-Myc [42]. In this study, we report that CPI-613 has the potential to upregulate GLS1, leading to metabolic reprogramming towards greater glutamine dependence, which represents a new mechanism by which cancer cells develop resistance to CPI-613.

As glutamine metabolism is enhanced in most cancers, many potent small-molecule inhibitors have been developed to target GLS1, including CB-839 and the selenadiazole-derivatives CPD-20, CPD-23, and physapubescin I [32, 43, 44]. CB-839, which was used in the present study, is known to have broad anticancer activity and currently remains under evaluation in dozens of clinical trials [32, 45, 46]. CB-839 selectively inhibits GLS1 but not GLS2, and as a monotherapy it has only a very limited anticancer effect [32]. Recently, CB-839 has been increasingly explored in combination approaches to treat cancer. For example, CB-839 can effectively overcome therapy resistance induced by the mTOR inhibitor MLN128 in preclinical animal models of lung squamous cell carcinomas [47]. Cabozantinib, a VEGFR2/MET/AXL inhibitor, is currently being investigated by our group in combination with pembrolizumab in the treatment of recurrent or metastatic incurable HNSCC (NCT03468218). Interestingly, the combination of cabozantinib and CB-839 has shown encouraging clinical activity and tolerability in patients with metastatic renal cell cancer compared to cabozantinib monotherapy [48]. Further exploration of the therapeutic potential of CB-839 in combination with cabozantinib for HNSCC treatment is warranted. Here, we uncover for the first time that CB-839 overcomes metabolic adaptation to CPI-613-mediated inhibition of lipoate-sensitive regulatory processes in HNSCC cells and demonstrate that the dual drug treatment exhibits a superior anti-HNSCC effect that may offer a potential novel therapeutic strategy.

## Conclusions

Coupled with rigorous in vitro and in vivo validations, our work not only reveals the critical role of GLS1-mediated glutaminolysis in CPI-613 treatment but also develops a promising anti-HNSCC regimen through the combination of CPI-613 and the GLS1 inhibitor CB-839. Our findings also suggest that alternative pathways of metabolism enable cancer cells to respond to metabolic stress and inhibiting the related compensatory pathways may achieve synergistic anticancer outcomes, laying a scientific foundation for developing more effective treatments targeting the metabolic requirements of tumor cells.

## Supplementary Information

**Additional file 1: Supplementary Fig. S1.** ATP amount in HNSCC cells treated with or without CPI-613 in culture medium supplemented with glucose or pyruvate. HN6 and HN12 cells were cultured in DMEM medium overnight, followed by addition of 100 μM CPI-613 and 25 mM glucose or 5 mM pyruvate. After 24 h of treatment, the amount of ATP in cell lysates was determined by CellTiter-Glo® 2.0 Assay Kit. *p < 0.05; **p < 0.01.

## Data Availability

All data generated or analyzed during this study are included in this published article [and its supplementary information files].

## Abbreviations

α-KGDH: Alpha-ketoglutarate dehydrogenase
ACC: Acetyl-coenzyme A carboxylase
AMPK: 5′ AMP-activated protein kinase
CI: Combination index
EGFR: Epidermal growth factor receptor
FCCP: Fluoro-carbonyl-cyanide phenylhydrazone
FITC: Fluorescein isothiocyanate
GAC: Glutaminase C
GLS: Glutaminase
GSH: Glutathione
HNSCC: Head and neck squamous cell carcinoma
HRs: Hazard ratios
IACUC: Institutional Animal Care and Use Committee
IOD: Integrated optical density
*i.p.*: Intraperitoneal injection
KGA: Kidney-type
MTT: 3-(4,5-Dimethylthiazol-2-yl)-2,5-diphenyltetrazolium bromide
NSG: NOD.Cg-*Prkdcscid Il2rgtm1Wjl/SzJ*
OCR: Oxygen consumption rate
Oligo: Oligomycin
OXPHOS: Oxidative phosphorylation
PDH: Pyruvate dehydrogenase
ROS: Reactive oxygen species
TCA: Tricarboxylic acid
TCGA: The Cancer Genome Atlas
