# Supplementary figures and images for: Blockade of glutamine-dependent cell survival augments antitumor efficacy of CPI-613 in head and neck cancer
Source: J Exp Clin Cancer Res. 2021 Dec 14;40:393. doi: 10.1186/s13046-021-02207-y (PMC8670127; doi:10.1186/s13046-021-02207-y)

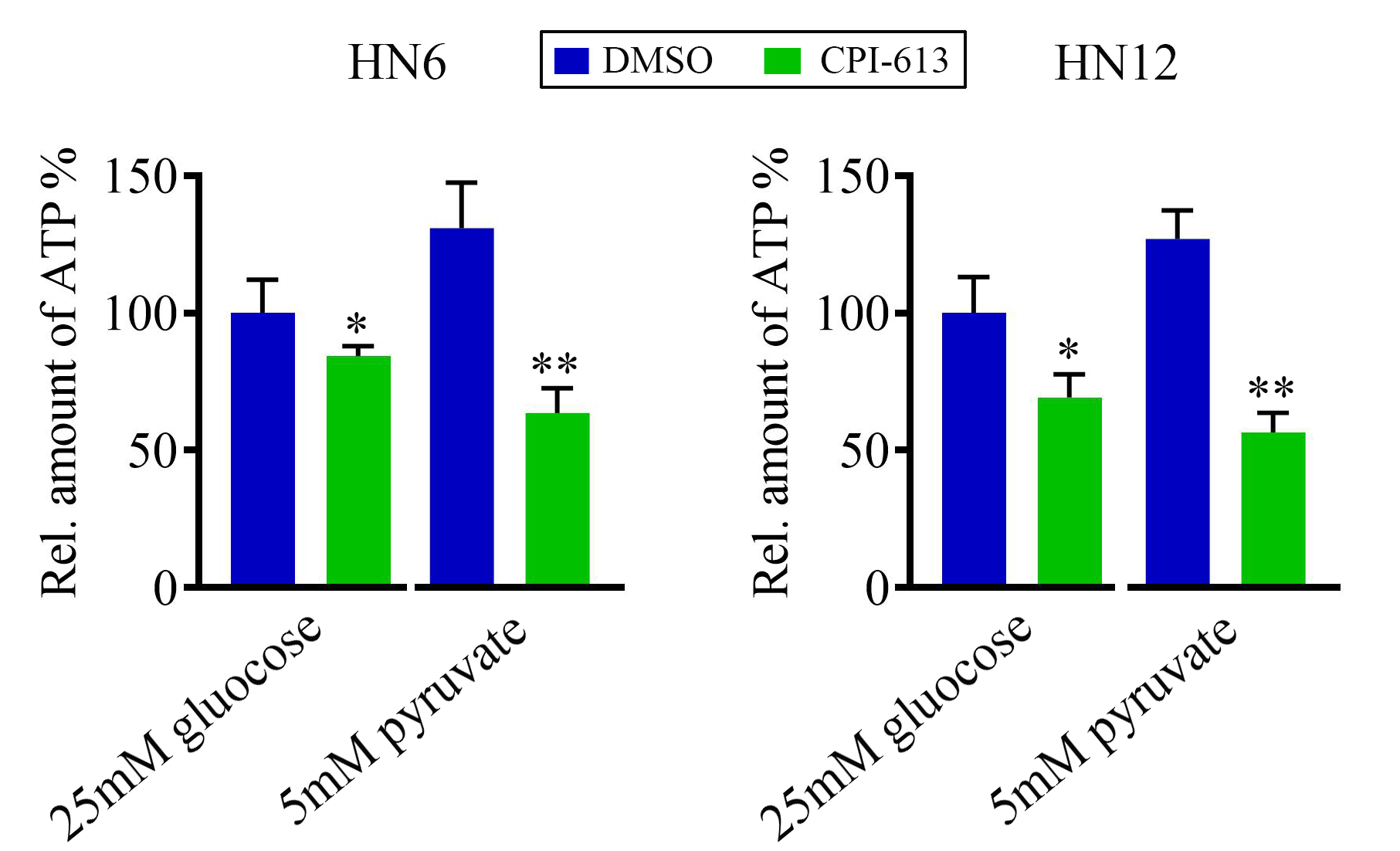

Supplement: Supplementary file 1 — Additional file 1: Supplementary Fig. S1. ATP amount in HNSCC cells treated with or without CPI-613 in culture medium supplemented with glucose or pyruvate. HN6 and HN12 cells were cultured in DMEM medium overnight, followed by addition of 100 μM CPI-613 and 25 mM glucose or 5 mM pyruvate. After 24 h of treatment, the amount of ATP in cell lysates was determined by CellTiter-Glo® 2.0 Assay Kit. *p < 0.05; **p < 0.01. [file 13046_2021_2207_MOESM1_ESM.tif]
